# Electronic personal health records for people with severe mental illness; a feasibility study

**DOI:** 10.1186/s12888-015-0558-y

**Published:** 2015-08-06

**Authors:** Dan Robotham, Matthew Mayhew, Diana Rose, Til Wykes

**Affiliations:** Department of Psychology, Institute of Psychiatry, Psychology & Neuroscience, King’s College London, De Crespigny Park, London, SE5 8AF UK; Department of Health Services and Population Research, Institute of Psychiatry, Psychology & Neuroscience, King’s College London, De Crespigny Park, London, SE5 8AF UK

**Keywords:** Personal health record, Self-monitoring, Self-management, e-health, Patient reported outcome measures

## Abstract

**Background:**

Electronic Patient Health Records (ePHRs) contain information created, accessed, monitored and maintained by patients. This paper describes how an ePHR called myhealthlocker™ was used by people with severe mental illness to monitor and input their own health-related outcomes, and whether they derived any benefit from it.

**Method:**

Individuals using local secondary mental health services were provided with access to myhealthlocker, an ePHR which allowed them to monitor their health and input information from Patient Reported Outcome Measures (PROMs) across to their clinical record. Participants were given support to use myhealthlocker through drop-in sessions facilitated by an Occupational Therapist. Usage of the site was monitored over time. Surveys and interviews were used to investigate what participants thought about the intervention.

**Results:**

32 of 58 participants used the ePHR (where usage was defined by logging in at least twice and completing a PROM). Almost all participants who used the site had been referred from community rather than inpatient services. Of those who used the site, 26 out of 32 used it primarily or exclusively through supported drop-in sessions. Almost half of those participants who used the site had used it outside the drop-in sessions. Those who used the site found it useful (n = 32), and most said they would continue to use it (n = 27). There were no apparent differences in usage across gender, diagnosis, and length of service use history. Suggestions for improvement included a social networking component, and finding ways to engage clinicians. In particular, users valued the ability to monitor health outcomes over time.

**Conclusions:**

People with severe mental illness were able to use an ePHR and derive benefit from monitoring and inputting PROMs. Those who use the site are more likely to have been referred from community mental health services, and then supported to access the ePHR.

## Background

An electronic Personal Health Record (ePHR) is edited, managed and owned by the patient. It is different to (and separate from) the patients’ clinical record, which is almost always controlled and managed by the healthcare provider and can be shared amongst clinicians as needed. Whilst ‘patient portals’ typically allow patients to access limited areas of their clinical record, an ePHR gives them complete control over the record, allowing them to share the information with clinicians as they wish.

Electronic Personal Health Records (ePHRs) are now represented in policy [1–3], the UK Government has called for *“a change in culture and mind-set, in which our health and care professionals, organisations and systems recognise that information in our own care records is fundamentally about us – so that it becomes normal for us to access our own records easily.”* [4].

Supporters of ePHRs claim that they enable continuity of care [5, 6], reduce medical errors [7], save resources [8], and allow consumers to become more informed about their health, which facilitates communication and empowerment [9, 10]. Nonetheless, ePHRs are a subject of public ambivalence [11], with difficulties in sustaining users’ interest over time [12]. Nowhere have problems been more evident than with the UK NHS HealthSpace, which was deemed difficult to use and not useful, and was discontinued following low rates of patient uptake. Among the criticisms were that end-users were not involved enough in development, and that it did not support patients to self-manage their health conditions [13].

One way in which ePHRs could be made more appealing is to enable users to input information into the clinical record [12]. This information could be self-monitored, personally relevant information (i.e., Patient Reported Outcome Measures; PROMs). Recent advances in Smartphone and wearable technology increase the potential interactivity of ePHRs; monitoring (and potentially, inputting) can be done in real-time [14] with a range of health outcomes including weight loss [15], dietary goals [16], smoking [17], diabetic control [18, 19], and blood pressure [20].

The use of ePHRs will depend upon their universal accessibility and adaptability. The focus of this paper is the usage of ePHRs amongst people with severe mental illness (SMI). There is evidence to suggest that computerised self-monitoring can be used to help people with depressive symptoms [21] and schizophrenia [22], and that ePHR systems have improved medical care and increased appropriate use of health services for people with SMI [23]. Nonetheless, health services have difficulty engaging this population (almost 60 % of patients with psychosis did not attend their first appointment following hospital admission [24]), and web-based information may not be accessible for people with SMI, especially for people with psychosis [25]. Such information must account for possible difficulties in processing information, concentration, lacking energy and experiencing paranoia [26].

The digital divide between people with SMI and the general population is closing [27]. This study describes the development of an ePHR system (myhealthlocker™) to help people with severe mental illness manage, monitor their health online and enter PROMs. The ePHR was developed with stakeholder involvement [28]. We aimed to engaging at least 50 % of those patients who were referred. This article reports how the ePHR was used in practice, and how stakeholders thought it should be improved.

## Method

### Setting/Overview of the ePHR

Before developing the ePHR, the research team conducted short interviews with 121 patients (100 of whom had experience of psychosis) within South London [27]. Findings suggested that older adults with psychosis wanted to improve their computer literacy, that people from black, minority and ethnic groups may need extra support when engaging with online health-related information, and that mobile phones and computers were the most familiar devices for accessing the Internet.

The website was developed within South London and Maudsley NHS Foundation Trust (SLaM). This covers a large, ethnically diverse population. Patients were referred to myhealthlocker by clinicians, either after exiting inpatient wards or through community services. Patients were invited to attend a facilitated ‘drop-in’ session at a local community venue, led by an Occupational Therapist. The purpose of these sessions was to enable patients to access the Internet, teach basic computer skills and introduce them to the features of the ePHR. Patients who attended drop-ins but who had no other way of accessing the Internet were lent mobile devices.

The myhealthlocker ePHR allows patients to monitor health-related outcomes and complete PROMs. These data are sent automatically to the clinical record, where they can be read both by patients and their clinicians.

### Design

This was a mixed methods longitudinal study to ascertain how people with SMI might use an ePHR. We defined whether or not they had used the ePHR based on the definition of completion of a self-monitoring outcome measure plus logging into the ePHR on two separate occasions. Using a mixture of community and inpatient settings, data were derived from three sources (1) self-report questionnaires, (2) auditing participants’ usage of the ePHR and (3) participants’ completion of PROMs. Data were supplemented by interviews with a smaller sample of patients who had used myhealthlocker. Participants were not obliged to allow research access to their medical record, therefore provided only anonymised data.

### Participants

Participants were recruited from community outpatient services (for people with psychosis) and from inpatient ‘triage’ units across South London. Care-coordinators within community services referred people into the programme. Link-workers within inpatient units would signpost participants to the external drop-in sessions, all participants recruited via this route were soon to be discharged from the ward. Interviews were conducted with ten patients.

### Measures

#### Usage

Login statistics were mapped for all individual users, with the date of each login and whether this was made ‘dependently’ (i.e., as part of a facilitated drop-in session) or ‘independently’ (outside of these hours).

‘Participants were asked to complete the Warwick Edinburgh Mental Wellbeing Scale (WEMWBS). This has been validated for measuring....Etc’

It has been validated for measuring mental wellbeing in people aged 16 years and over in the UK, and has proved popular with practitioners, policy-makers and service-users [29].

#### Feasibility and acceptability

A 17-item feasibility questionnaire about the experience of setting-up an account and using the site, PROMs completion and views on the speed, design, and layout of the site. The survey was predominantly quantitative, including yes/no and 5-point Likert scales, but there was one open-ended question on how the site could be improved. Some participants wrote additional comments on the questionnaire form. The questionnaire included one item on patients’ computer confidence prior to using myhealthlocker to provide some context for the answers.

Qualitative interviews were conducted using a topic guide, developed in partnership with the myhealthlocker team. The topic guide included questions on whether the ePHR had been useful, what patients most liked about the site, whether they would recommend the site to others, whether they had shared the results of their health monitoring with their clinician(s), concerns about confidentiality, and what could be improved.

#### Context

Demographic information was collected on participants’ age, ethnicity and history of service usage. Diagnostic information was extracted through participants’ clinical records (in cases where the participant had provided informed consent for the researcher to do this).

### Procedure

The project team gained approval from an independent Research Ethics Committee (NRES Committee London - Camden & Islington, Reference 10/H0722/79). Participants were given personal login information to access myhealthlocker. After completing at least one WEMWBS, participants were asked to complete the feasibility survey and a sub-sample participated in face-to-face qualitative interviews after they had been using with the site for a prolonged period, typically three months. The period of data collection was 28/02/2013 to 11/07/2014.

### Definitions of usage

We defined a person having ‘used’ myhealthlocker as logging in at least twice and completing at least one WEMWBS survey. The completion of a WEMWBS survey indicated that patients had used the self-monitoring function of the site and were able to provide feedback on it. This is a stringent criterion as many individuals may have only wanted to browse the online services; information on medication, health conditions and disability benefits.

Patterns of usage were explored to identify instances of ‘dependent usage’. These were coded as occurring within the drop-in session, 10 min before or 30 min afterwards. Using the site outside these parameters was coded as ‘independent’. Participant attendance at drop-ins each week was corroborated using attendance registers. Separate logins within two hours of each other were conflated in order to account for unintentional logouts or for multiple logins in a short space of time. Two researchers independently classified each login as ‘dependent’ or ‘independent’ and discussed any disagreements.

### Sample size

No formal sample size calculation was conducted; we recruited participants as they were referred from local services to use myhealthlocker. The aim was to recruit at least 50 individuals and monitor their usage over a 12 month time period.

### Data analysis

Participants’ usage with the site was measured as the primary outcome. For those who used the ePHR, we analysed participant feedback using descriptive statistics. Secondary analyses focused on the factors that may relate to usage using two-sided chi-squared tests. Qualitative data from the questionnaire and interviews were analysed using constant comparison. Two researchers devised a coding frame, and then analysed the data in accordance with this frame. Initially the coding frame was based upon the topic guide, but this was revised whilst coding the data to account for new themes arising. The second iteration of the coding frame was agreed by the coders after analysing half of the interviews. The coding frame was then revised following analysis of all ten interviews. The main themes were agreed between the two coders (DR, MM). A similar mixed methods analysis strategy has been used in other studies which have investigated website and app usage in the health field [30].

## Results

### Sample characteristics

The overall sample included 58 people. Sample characteristics are shown in Table 1, 34 participants were recruited from community services and 24 from inpatient services prior to community discharge. 49 participants gave the research team permission to access their clinical records data (which included their self-entered WEMWBS data). The demographic characteristics represent the population of people currently engaged with services for people with severe mental illness services.

**Table 1:** Demographic and clinical characteristics of participants

Variable	Engaged Respondents		Non-Engaged Respondents		Semi-structured interviews
	*N* (Total = 32)	%	*N* (Total = 26)	%	*N* (Total = 10)
*Age (years)*					
18-24	1	3	5	19	0
25-34	2	6	9	35	2
35-44	14	44	3	11	3
45-54	8	25	8	31	3
55-64	7	22	1	4	2
65+	0	0	0	0	0
*Gender*					
Male	19	59	15	58	5
*Ethnicity*					
White	15	47	11	42	4
Any mixed background	3	10	2	8	3
Asian/Asian British	2	6	1	4	1
Black/Black British	10	31	11	42	1
Chinese/Any other ethnic group	2	6	1	4	1
*Length of time in contact with mental health services*					
1 year or less	6	19	8	31	1
2-5 years	9	28	6	23	3
6-10 years	3	9	7	27	1
More than 10 years	14	44	5	19	5
*Primary diagnosis*					
Psychosis and related	10	31	11	42	2
Mood or anxiety disorder	7	21	7	27	4
Personality disorder	1	3	3	12	1
Substance abuse	0	0	2	8	0
No diagnosis	1	3	0	0	0
Unknown/no consent	13	41	3	12	3

### Usage

In total, 32 participants used the ePHR. The majority of these participants had been referred through community services with only one referred from an inpatient unit (χ =43.1, df = 1, sig < .001).

Although younger people (aged below 35) appeared less likely to use myhealthlocker than those aged 35, this was confounded by younger participants often being referred from inpatient units. When considering only those who were recruited from the community (n = 34), there were no demonstrated usage differences by age (χ = 3.5, df = 4, sig < .47). Usage did not differ by gender (Fisher Exact test = .017, df = 1, sig = 1), by diagnosis (Fisher Exact test = .096, df = 1, sig = 1), length of time using services (one year or less versus longer service history (n = 44; Fisher Exact test = 1.1, df = 1, sig = .36).

Login data were available from 32 participants who had used with the site. Of these, there were 1156 logins within the data collection period (after adjustment for multiple logins and unintentional logouts) (individual data: min = 2, max = 476, mean = 36). Of these, 514 logins occurred within the drop-in sessions and 642 occurred independently. Most of the participants who used the site (n = 26 out of 32) used the site primarily or exclusively at the facilitated drop-ins. Although 19 people had used the site outside of drop-ins, one participant accounted for 433 (67 %) of all independent logins. If this ‘outlier’ is removed then 31 % of logins (*n* = 209 of 680) occurred independently of drop-in sessions.

In order to gauge usage patterns over time, we analysed only those participants who had been using the ePHR for one year prior to the end of the data collection period (n = 17). For these participants, all had used the site for three consecutive months or more. On average, participants used the site for ten months (min = 3, max = 18). Five had used the site within the last two months of data collection; the remaining 12 appeared to have stopped using the site.

Amongst participants who consented to allow access to their PROM data (n = 27), 466 PROMs had been completed. This represents an average of 17 per participant (min = 1, max = 65). The majority of PROMs (n = 380, 82 %) had been completed at drop-in sessions, although most participants (19 out of 27) had completed it at least once independently.

### Feasibility and acceptability

According to participants’ responses to the feasibility survey (n = 32), all but one said that they found the site useful, and 27 thought they would continue to use it in the future. The majority (n = 22) reported that they were confident in using computers prior to using myhealthlocker. The majority (n = 24) also said the login process was ‘simple’ (only four found it ‘complicated’). Participants reported few problems with the layout of the site (mean = 2.4, SD = 1.5), the text size (mean = 1.8, SD = 1.5), navigating the site (mean = 2.7, SD = 1.9) or understanding the content (mean = 1.8, SD = 1.5). Each of the above had been measured on 5 point Likert scales where a lower score represents a more positive response. Additional comments referred to limitations in how the results of the PROM were presented; *“the graph a bit difficult to interpret”*. One patient commented that the site should be linked to primary care; *“good to be able to access GP and health records in future”*.

For those completing the wellbeing PROM there was improvement when comparing participants’ last completed PROM (mean = 45, SD = 14) against their first completed PROM (mean = 40, SD = 9). These differences were significant using paired *t*-test (t = −2.6, df = 26, 2-sided sig = .016). The scale can therefore detect variability over time, and anecdotal information suggests that some participants were able to use this variability in noting changes in their use of treatment or events in their life. 20 individuals with completed PROMS had looked at the survey results. Most reported little difficulty in completing the PROM, rating it a mean of 1.5 on a scale of 1–5 (where 1 is defined as ‘simple’ and 5 as ‘complicated’). Most found the PROM questions relevant to them (mean = 2.2 out of 5), and did not feel that the process of completing the PROM was time consuming (mean = 2 out of 5).

Of those who took part in the qualitative interviews (n = 10), all said that they would recommend myhealthlocker to others and some had done so. The ‘interactive’ parts of the site were most valued. All respondents liked the self-monitoring aspect, most mentioned it unprompted. One participant described the process of completing PROMs as difficult but worthwhile.

*“It's useful being able to monitor my mood over the weeks and months.” (Participant #4)*

*“you can track that over the weeks and see like how you’ve generally been doing and when there have been good times for you and when has been bad.” (Participant #6)*

*“I can see…for myself what’s making me feel bad, what’s making me feel good, whereas before I had nothing to go by; so it was just a word of my care team.” (Participant #3)*

*“I’m pushing myself to do it.” (Participant #1)*

The facilitated drop-in sessions provided patients with opportunities to socialise. When an online social networking feature was suggested, several participants thought this was a good idea, but some expressed concerns about linking it to an existing social network such as Facebook.

*“The Edinburgh Wellbeing Score to do every week is quite good because you can then discuss it with other people and realise what score you are in relation to other people.” (Participant #5)*

*“It would be cool if there was a kind of source of connectivity on there as well.”(Participant #6)*

*“I don’t know that I particularly would want it popping-up on my Facebook profile, that I was a user of myhealthlocker, I don’t know. […] I would want total control over it.” (Participant #2)*

Some were using their PROM results to inform clinicians. For others, lack of clinical engagement was a source of frustration. One participant had never considered the idea of giving PROM results to his clinician.

*“At least now, my CBT therapist can access it and see exactly how I’m feeling.” (Participant #3)*

*“So my care team can know where and what progress, or I’m not making any progress how to handle the situation.” (Participant #10)*

*“I would appreciate it if both the psychiatrist and the GP were interested to be, you know, to look at it.” (Participant #2)*

*“This concept never came to my mind […] myhealthlocker is for my own use.” (Participant #8)*

## Discussion

The study demonstrates that people with severe mental illnesses can use ePHR systems. Improvements in wellbeing over time and comments from interviewees suggest potential benefit of using ePHRs to self-monitor health outcomes. Using the system depends on the context in which the patient is introduced to it; our data suggest that patients are unlikely to use the site if they are not given support to help them use it. However, if referred through community services and provided with support, patients are likely to use the site over a sustained period. Patients in this study were able to access both technical and clinical help. This included computer skills training, connecting to the internet, navigating the ePHR, entering patient reported outcomes and setting goals. Previous studies allude to the need for technological support for people with severe mental illness [31] such as support for entering and retrieving data from ePHRs [23], but there is growing evidence for the use of remote computerised therapies in this population [32], which is likely to become more prevalent in future.

The success of a self-monitoring ePHR depends on patient’s willingness to use the application (and complete PROMs) independently. Our findings suggest that online self-monitoring is feasible and acceptable, and that some people will use the site independently on a regular basis. A previous US study suggested it was possible to engage similar client groups in technological applications using daily automatic prompts, though site users were also paid for the time spent on study assessments [22] which was an additional inducement. Our experience suggests that some form of support is needed even if individuals suggest that they are confident with computers.

Analysis of qualitative interviews reveals a desire for greater connectivity and interactivity. This may underpin why patients preferred to complete PROMs at weekly drop-in sessions rather than independently. At present, myhealthlocker does not support an interactive ‘social networking’ component. Such a development has appeal but should be approached with caution, reduced social networks may predate psychotic symptoms [33], and online networks may help people with psychosis cope with isolation [34]. Nonetheless, young adults with psychosis can be vulnerable to the pitfalls of online social media [35].

This study provides longitudinal information on how people with severe mental illnesses use an ePHR over time. This is triangulated with qualitative information from those who have used the site, showing how the site could be developed in future, and describing the interactive elements which patients valued. The relatively high level of usage amongst participants (32 out of 58) is one of the highest achieved in the literature. The study is limited by focusing only on those who used the intervention, as fewer useful data were available for participants who did not use the site or attend drop-in sessions. We have preliminary evidence to suggest that people use myhealthlocker over an extended period, but we do not know whether people would continue to use the ePHR in the absence of facilitated support. Future research should focus on how support should be given to help people use the site, how clinicians might engage to provide this support, and whether site users benefit in the long-term.

## Conclusions

For parity with physical health, it is essential that people with severe mental illness are offered real opportunity to use digital innovations that can help them manage and monitor their health if they wish to do so. Identifying and removing the barriers to engagement was the purpose of this study. Individuals valued the ability to complete PROMs and monitor health over time, but often required support in order to do this. For services this means an increased initial cost for drop-in clinics for patient and clinician training and support. This is the first step in embedding digital health in a mental health service. We have not reported on clinician engagement which will be our next focus. What was clear from our data is that patients would prefer clinicians to engage, even if clinicians are not currently enthusiastic.

## Abbreviations

BME: Black and Minority Ethnic
ePHR: electronic Patient Health Record
NHS: National Health Service
NRES: National Research Ethics Committee
PROM: Patient Reported Outcome Measure
SLaM: South London and Maudsley NHS Foundation Trust
SMI: Severe Mental Illness
WEMWBS: Warwick-Edinburgh Mental Well-Being Scale
